# Effects of Two Distinct Psychoactive Microbes, *Lacticaseibacillus rhamnosus* JB-1 and *Limosilactobacillus reuteri* 6475, on Circulating and Hippocampal mRNA in Male Mice

**DOI:** 10.3390/ijms23179653

**Published:** 2022-08-25

**Authors:** Sandor Haas-Neill, Eiko Iwashita, Anna Dvorkin-Gheva, Paul Forsythe

**Affiliations:** 1 The Brain Body Institute, St. Joseph’s Hospital, McMaster University, Hamilton, ON L8N 4A6, Canada; 2 McMaster Immunology Research Centre, Department of Medicine, McMaster University, Hamilton, ON L8N 3Z5, Canada; 3 Division of Pulmonary Medicine, Department of Medicine, University of Alberta, 569 Heritage Medical Research Center, Edmonton, AB T6G 2S2, Canada; 4 Alberta Respiratory Centre, University of Alberta, Edmonton, AB T6G 1H9, Canada

**Keywords:** depression, stress, gut–brain-axis, JB-1, psychobiotics, miRNA, mRNA, hippocampus, blood, microbiota

## Abstract

Discovery of the microbiota-gut–brain axis has led to proposed microbe-based therapeutic strategies in mental health, including the use of mood-altering bacterial species, termed psychobiotics. However, we still have limited understanding of the key signaling pathways engaged by specific organisms in modulating brain function, and evidence suggests that bacteria with broadly similar neuroactive and immunomodulatory actions can drive different behavioral outcomes. We sought to identify pathways distinguishing two psychoactive bacterial strains that seemingly engage similar gut–brain signaling pathways but have distinct effects on behaviour. We used RNAseq to identify mRNAs differentially expressed in the blood and hippocampus of mice following *Lacticaseibacillus rhamnosus* JB-1, and *Limosilactobacillus reuteri* 6475 treatment and performed Gene Set Enrichment Analysis (GSEA) to identify enrichment in pathway activity. *L. rhamnosus*, but not *L. reuteri* treatment altered several pathways in the blood and hippocampus, and the *rhamnosus* could be clearly distinguished based on mRNA profile. In particular, *L. rhamnosus* treatment modulated the activity of interferon signaling, JAK/STAT, and TNF-alpha via NF-KB pathways. Our results highlight that psychobiotics can induce complex changes in host gene expression, andin understanding these changes, we may help fine-tune selection of psychobiotics for treating mood disorders.

## 1. Introduction

Anxiety and depression are two of the most common mood disorders in the western world becoming increasingly prevalent in millennials and adolescents [1,2]. The COVID-19 pandemic only exacerbated the problem. Among adults in the UK the reported rate of depression symptoms nearly doubled from pre-to-post pandemic (10% to 19%), and in the US it nearly quadrupled (11% to 42%) [3]. Much attention has been given to the gut–brain-axis in recent years as it is beginning to revolutionize our understanding and treatment of mental health disorders [4].

Numerous direct and indirect interactions between bacteria endemic to the gut, and the central nervous system characterize what is known as the microbiota-gut–brain axis [5]. The introduction of certain bacteria to the gut that modulate brain function, termed psychoactive-probiotics or psychobiotics, have been demonstrated to influence behaviour in animal models and mood/anxiety in humans [6,7]. One such potential psychobiotic is *Lacticaseibacillus rhamnosus* JB-1, which has previously been shown to reduce anxiety and depression-like behaviours in mice [8,9,10].

It is incompletely understood how JB-1 facilitates these cognitive and behavioural changes, although both the peripheral nervous system and the immune system are critical mediators [8,9,11]. Specifically, feeding of JB-1 was only able to alleviate the depression and anxiety-like behaviours of mice when the vagus nerve was intact [9]. Feeding of JB-1 also results in modulation of the immune system and induces regulatory T cells, which have been demonstrated to be both necessary and sufficient to mediate the behavioral effects of the bacteria [8,11]. Other psychobiotics have been suggested to modulate behaviour and cognition via the endocrine system, the release of soluble metabolites including neurotransmitters into circulation, and the release of bacterial membrane vesicles (MV) carrying similar metabolites and RNAs into circulation [12,13,14,15]. Up to this point however these mechanisms have not been demonstrated to be involved in the behavioural changes induced by *L. rhamnosus* JB-1.

*Limosilactobacillus reuteri* 6475 (LR6475) is a probiotic bacteria that has previously been shown to modulate social behaviours; rescuing autism-spectrum-disorder-like social deficits induced by a maternal high-fat diet in mice [16]. Mechanistically, LR6475 achieves this in a vagus-dependant manner and by boosting oxytocin levels [17,18]. LR6475 has also been shown to have efficacy in treating irritable bowel syndrome and increase bone density via T-lymphocyte regulation [19,20]. However, despite both JB-1 and LR6475 engaging the vagus nerve and regulatory immune responses the bacteria have some distinct actions on behaviour with JB-1, but not LR6475, having antidepressant-like effects in mice [21]. The reasons for the distinct behavioral effects of the bacteria is unclear.

Here, in an attempt to identify potential pathways distinguishing two psychoactive bacterial strains that seemingly engage similar gut–brain signaling pathways but have distinct effects on behaviour, we compare transcriptomic changes in blood and hippocampus, a region of the brain responsible for memory and emotion and closely linked with depression [22,23,24], following feeding with JB-1 and LR6475.

## 2. Results

### 2.1. Many mRNAs and Gene Sets Are Altered in the Blood of JB-1-Fed Mice, but Not LR6475-Fed

**Figure 1:**
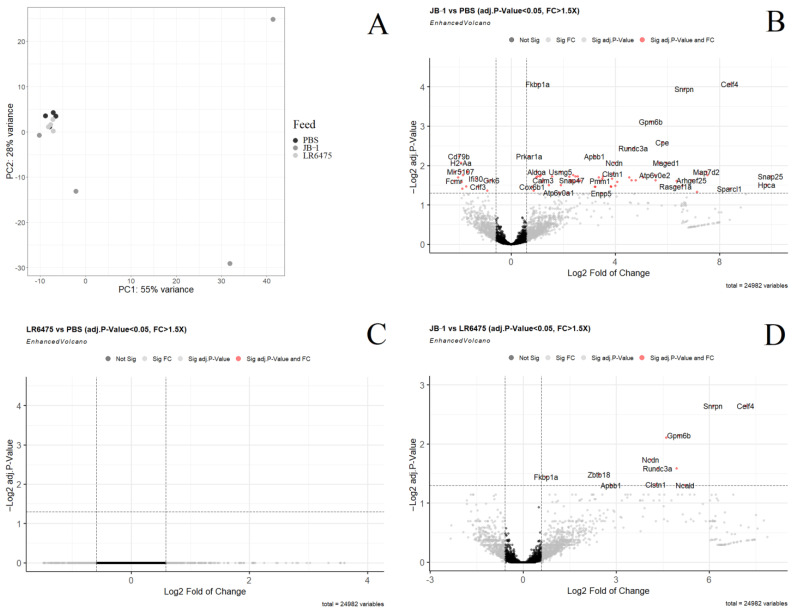
Differential expression analysis of mRNA measured by RNAseq in the blood of mice fed either *Lacticaseibacillus rhamnosus* JB-1, *Limosilactobacillus reuteri* 6475, or PBS. (**A**) Principal component analysis of all three treatment groups. (**B**) Volcano plot showing individual differentially expressed genes in a comparison of JB-1 vs. PBS-fed mice. (**C**) Volcano plot showing individual differentially expressed genes in a comparison of LR6475 vs. PBS-fed mice. (**D**) Volcano plot showing individual differentially expressed genes in a comparison of JB-1 vs. LR6475-fed mice. Genes with a positive log fold change are more highly expressed.

Principal component analysis (PCA) of normalized, filtered mRNA in the blood of mice shows no distinct groups between PBS and LR6475 fed mice; however, JB-1 fed mice differ greatly from the cluster PBS and LR6475-fed mice form along both PC 1 and 2 (adonis *p*-value = 0.049) (Figure 1A). As the PCA indicates, many genes were found to be differentially expressed when comparing JB-1 to PBS-fed mice (Figure 1B) and a few-when comparing JB-1 to LR6475 (Figure 1D); however, no genes were significantly differentially expressed between LR6475 and PBS-fed mice. FKBP1A was among the genes upregulated in the blood of JB-1-fed mice in both comparisons.

To elucidate additional sources of grouping along PC1 and 2 in the blood, Gene Set Enrichment Analysis was performed, comparing KEGG and Hallmark gene set expression in JB-1-fed mice to LR6475 and to PBS-fed independently. LR6475 and PBS-fed mice were not compared as their groups were not distinct in PCA. Many pathways from both KEGG and Hallmark were found differentially expressed in both directions, in both JB-1 comparisons, and are summarized in (Table 1). All of the pathways enriched in JB-1 vs. PBS were common to the JB-1 vs. LR6475 comparison, with the exception of KEGG_LEUKOCYTE_TRANSENDOTHELIAL_MIGRATION, and KEGG_NATURAL_KILLER_CELL_MEDIATED_CYTOTOXICITY which were enriched in PBS compared to JB-1 but did not appear significant in the JB-1 vs. LR6475 comparison. 1 Hallmark pathway and 4 KEGG pathways were commonly enriched in JB-1 for both comparisons, and 6 Hallmark and 8 KEGG pathways were commonly enriched in LR6475, and PBS compared to JB-1. Mouse gene names converted to human orthologs were used to visualize KEGG pathways in pathview-these figures were created for the blood mRNA JB-1 vs. PBS comparison and can be found in the supplementary figures (Supplementary Figures S1–S18).

### 2.2. Few mRNAs and Gene Sets Are Altered in the Hippocampus of Psychobiotic-Fed Mice

**Figure 2:**
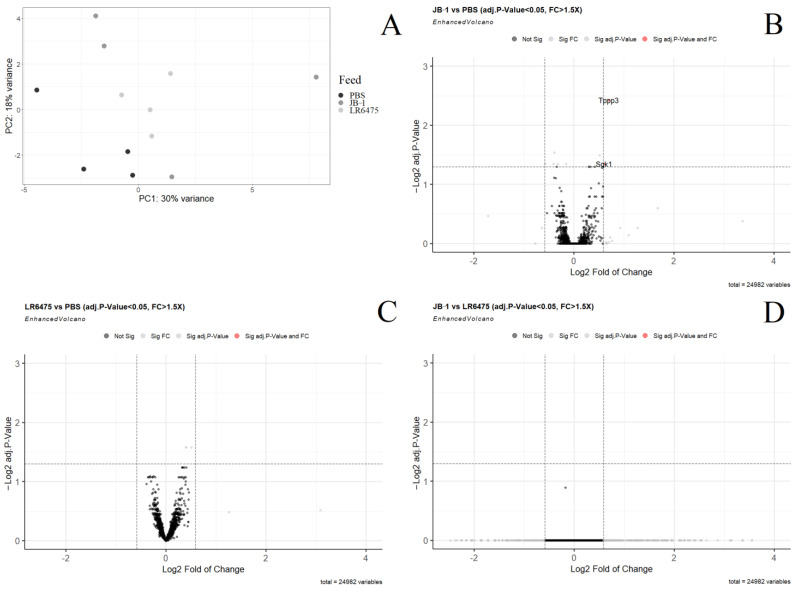
Differential expression analysis of mRNA measured by RNAseq in the hippocampus of mice fed either *Lacticaseibacillus rhamnosus* JB-1, *Limosilactobacillus reuteri* 6475, or PBS. (**A**) Principal component analysis of all three treatment groups. (**B**) Volcano plot showing individual differentially expressed genes in a comparison of JB-1 vs. PBS-fed mice. (**C**) Volcano plot showing individual differentially expressed genes in a comparison of LR6475 vs. PBS-fed mice. (**D**) Volcano plot showing individual differentially expressed genes in a comparison of JB-1 vs. LR6475-fed mice. Genes with a positive log fold change are more highly expressed.

In the principal component analysis of normalized, filtered mRNA in the hippocampus of mice, JB-1 and LR6475-fed do not form distinct clusters from one another; however, both JB-1 and LR6475-fed mice differ from the PBS cluster formed along both PC1 and 2 (adonis *p*-value = 0.17) (Figure 2A). Only 2 genes, TPPP3 and SGK1 were found to be differentially expressed when comparing JB-1 to PBS-fed mice (Figure 2B). When comparing JB-1 to LR6475, and LR6475 to PBS-fed mice (Figure 2C,D); however, no genes were found to be significantly differentially expressed. In order to identify sources of the group distinction for both PBS comparisons, Gene Set Enrichment Analysis was performed.

Enrichment analysis of hippocampal mRNA following treatment was also poorly able to identify the source of PCA group distinction. No Hallmark pathways were found differentially expressed in any of the three comparisons. KEGG_RIBOSOME was enriched in the hippocampi of both JB-1 (adj.*p*-value = 1.3 × 10^−4^) and LR6475-fed mice (adj.*p*-value = 2.4 × 10^−7^) compared to PBS-fed mice, and KEGG_VIBRO_CHOLERAE_INFECTION was enriched in PBS compared to LR6475 (adj.*p*-value = 1.6 × 10^−2^). With so few discerning features between individual gene expression and enrichment analysis, it remains unclear what is driving the distinction seen between PBS and the other treatment groups in the PCA. Additional PCs were checked (up to PC12) but none alone explain the distinction.

### 2.3. Weighted Correlation Network Analysis Confirms Differences in Blood Expression between Feeding Groups

**Figure 3:**
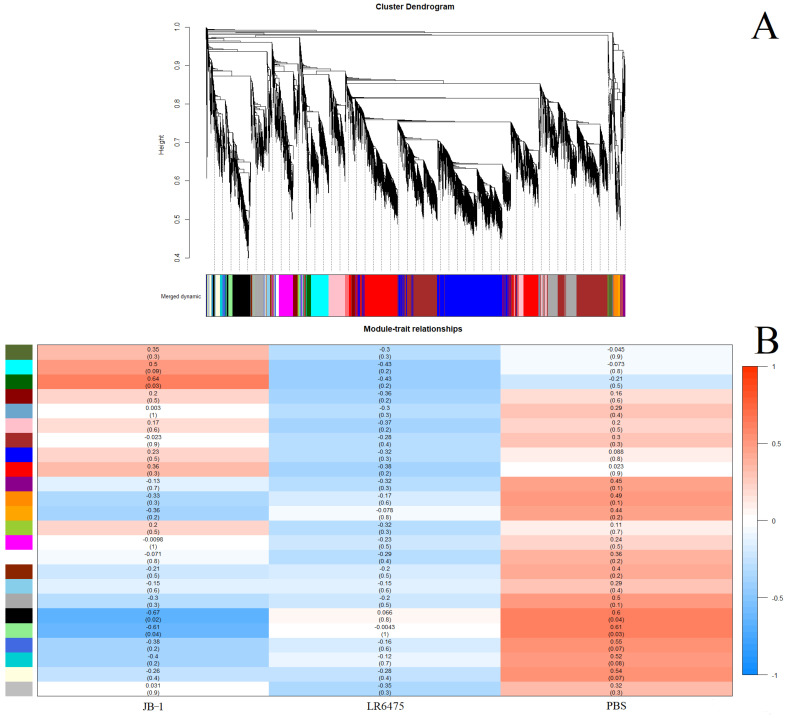
Weighted correlation network analysis (WGCNA) of blood mRNA from mice fed one of JB-1, LR6475, or PBS. (**A**) Unsupervised cluster dendogram of commonly co-expressed genes shown grouped into modules by colour. (**B**) Gene module and feed group relationships shown in a heatmap with relative expression level and *p*-value in brackets under it for each relationship.

1. As a second line of evidence that JB-1, but not LR6475 has a unique impact on blood and hippocampal mRNA expression, we performed weighted correlation network analysis (WGCNA) which relies on unsupervised clustering of genes to construct a network with modules of commonly co-expressed genes.
2. Using a power of 13 for blood and merging at a threshold of 0.05, 24 eigengenes were identified and mapped to the cluster dendogram (Figure 3A). There were several strong relationships identified when comparing these gene modules to the feed groups (traits) including statistically significant differences between JB-1 and PBS-fed mouse blood in the ‘black’ and ‘light green’ groups (Figure 3B). None of the modules had a significant relationship with LR6475-fed mice, making it statistically indiscernible from PBS, which is consistent with the PCA (Figure 1A).

**Figure 4:**
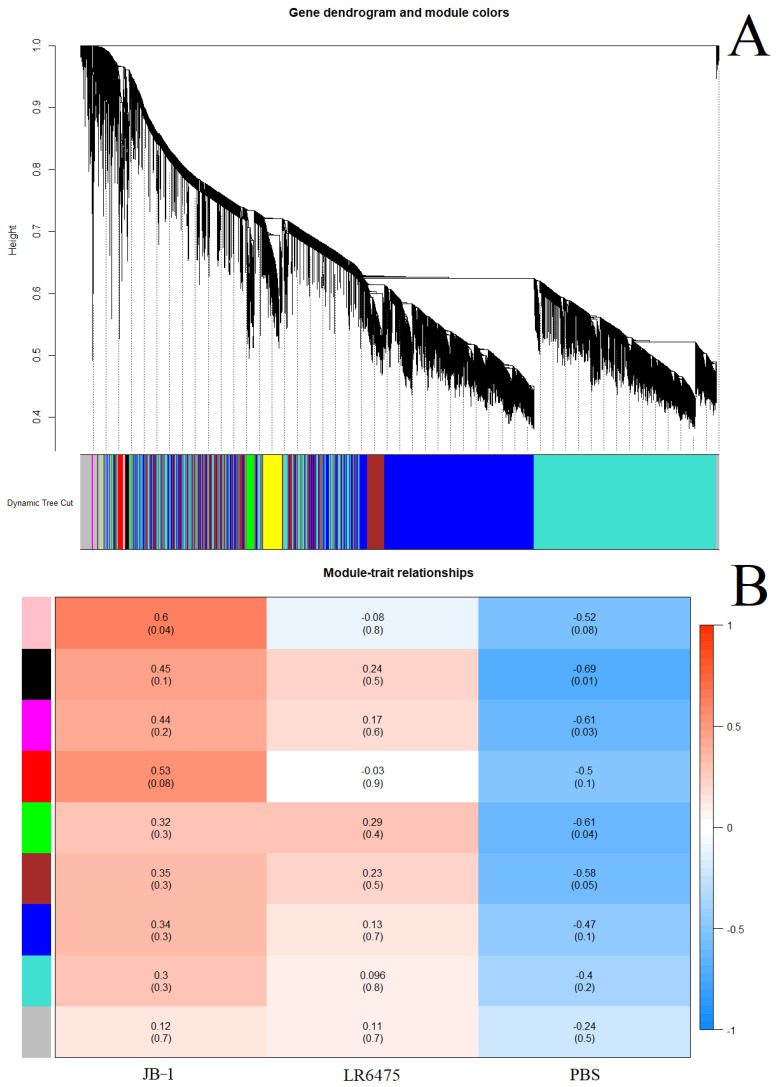
Weighted correlation network analysis (WGCNA) of hippocampal mRNA from mice fed one of JB-1, LR6475, or PBS. (**A**) Unsupervised cluster dendogram of commonly co-expressed genes shown grouped into modules by colour. (**B**) Gene module and feed group relationships shown in a heatmap with relative expression level and *p*-value in brackets under it for each relationship.

For hippocampal mRNA, a power of 30 was used, and merging at a threshold of 0.04, 9 eigengenes were identified and mapped to a cluster dendrogram (Figure 4A). No statistically significant relationships between gene modules and feed group pairs were found (Figure 4B), which is consistent with the PCA (Figure 2A). Additionally, consistent with the PCA in Figure 2A is that JB-1 and LR6475 appear harder to distinguish on the heatmap, while PBS-fed mice appear distinct. There were also statistically significant associations between individual feed groups and gene modules: JB-1 was associated with pink, while black, magenta, and green were associated with control. Again, LR6475 was not significantly associated in either direction with either gene module.

## 3. Discussion

Here, we examined blood and hippocampal transcriptional changes induced by two lactobacillus species that have previously been demonstrated to have distinct effects on behaviour in mice [8,9,10,16,21]. This study identified clear transcriptomic changes in the blood, and to a lesser extent, the hippocampus following feeding with JB-1, but not LR6475.

### 3.1. Inflammatory Response

Immunomodulatory actions have been described for both JB-1 and LR6475 and in the case of JB-1 these have been demonstrated to mediate effects on behaviour [8,9,10,16]. The current study identified transcriptomic changes reflective of immunomodulation. In particular, several genes involved in antigen presentation were enriched in the PBS and LR6475 vs. JB-1 (Figure 1B,D). Histocompatibility 2, class II antigen A, alpha (H2-Aa), a subunit of the major histocompatibility complex II (MHCII) enables peptide antigen binding activity, and it participates in the interferon-γ response [25]. Additionally, enriched in the blood of PBS and LR6475 vs. JB-1 was gamma-interferon-inducible lysosomal thiol reductase (IFI30) an enzyme that reduces endocytic disulphide bonds to bring about production of MHC class II-restricted epitopes [26,27]. B-cell antigen receptor complex-associated protein alpha chain and beta chain (CD79a and CD79b) were both enriched in the blood of PBS and LR6475 compared to JB-1-fed mice. These proteins cooperate, and are required for antigen presentation on B cells, as they facilitate the signal transduction cascade activated by an antigen binding to the B cell antigen receptor complex [28,29]. There are several lines of evidence suggesting JB-1 has physiological effects similar to selective serotonin reuptake inhibitors (SSRIs) [10,21,30]. It is therefore interesting to note that SSRIs such as fluoxetine have also been shown to modulate antigen presentation, reducing co-stimulatory marker expression on dendritic cells and subsequent antigen induced T cell response [31].

We also observed marked changes in mRNA related interferon signaling with significant diminution of HALLMARK_INTERFERON_ALPHA_RESPONSE, HALLMARK_INTERFERON_GAMMA_RESPONSE, and HALLMARK_INFLAMMATORY_RESPONSE in the blood of mice treated with JB-1 in comparison to both PBS and reuteri 6475. The Interferons are known to play a role in the link between the immune system and mood disorders. INF-α is used to treat hepatitis C and is associated with a 30–70% increased risk of emergent depression [32]. Interferon gamma (INF-γ) is also indicated to play a role in depression [33]. Patients with MDD demonstrate higher levels of INF-γ production by peripheral blood mononuclear cells [34] and successful antidepressant treatment decreases levels of this inflammatory cytokine while increasing regulatory IL-10 [35]. Furthermore INF-γ −/− mice demonstrate decreased anxiety- and depressive-like behaviors. More broadly, inflammatory cytokines including INF-γ are upregulated as part of the stress response, which in turn leads to activation of the microglia, hypothalamic-pituitary-adrenal (HPA) axis, and the sympathetic nervous system (SNS) [33]. Our observation of decreased INF-α, INF-γ, and inflammatory pathway activity in the blood, indicates a broad anti-inflammatory effect of JB-1 and is consistent with our previous findings of increased regulatory T cells and inhibition of mast cell degranulation [8,36,37]. Additional evidence for a general anti-inflammatory response to JB-1 is indicated by the decreased expression of the KEGG_T_CELL_RECEPTOR_SIGNALLING_PATHWAY in the blood of JB-1 treated mice compared to both LR6475 treated and control animals. The T cell receptor signaling pathway is critical for the activation of T lymphocytes (CD25+) which have previously been found elevated in the blood of depressed individuals [38]. The JB-1 associated differences in circulating gene expression of interferon signaling pathways was not observed in the hippocampus.

HALLMARK_TNFA_SIGNALLING_VIA_NFKB was underexpressed in both JB1 blood comparisons. These are genes that are regulated by NF-KB in response to TNF-α. TNF-α signaling through NF-KB has previously been shown to activate microglia and increase neuroinflammation in mice showing depression-like behaviour [39]. Mice instilled with depressive-like behaviour via chronic unpredictable mild stress also showed heightened levels of inflammatory cytokines, and NF-KB in the prefrontal cortex and hippocampus, the signaling of which was associated with greater risk of depressive symptoms [40].

Finally, FKBP12, one of the genes increased in expression in the blood of JB-1-fed animals compared to the other treatment groups, is a known inhibitor of mTOR signaling [41]. This may be part of the anti-inflammatory response to JB-1, as mTOR controls immune cell activity as well through assisting the differentiation of T cells, and by regulating translation, modulating cytokine responses, macrophage migration and polarization, and antigen presentation [41,42,43,44].

### 3.2. Cerebral Cortical Signaling

The Janus kinase/signal transducers and activators of transcription (JAK/STAT) signaling pathway controls several processes in the cerebral cortex and hippocampus, including microglial activation, synaptic plasticity, gliogenesis, and neurogenesis [45,46,47]. Here, we found it downregulated in the blood mRNA for both the Hallmark and KEGG gene sets (HALLMARK_IL6_JAK_STAT3_SIGNALING, KEGG_JAK_STAT_SIGNALING_PATHWAY). Al-Samhari et al. (2016) [48] found that treating rats with anti-oxidant precursor, N-acetylcysteine, inhibited STAT3 protein activation led to reduced depression-like symptoms in rats (increased locomotor activity). This is one example of why it has been proposed that JAK/STAT pathway inhibitors could serve as good candidates for antidepressants [47,48]. The reduced expression of the JAK/STAT pathway in the blood of mice after JB1 feeding suggests this may be a previously unrecognized mechanism contributing to the antidepressant-like effects of the bacteria.

Within all comparisons made, only two genes were significantly overexpressed in the hippocampus-TPPP3 and SGK1 which were altered in the JB-1 vs. PBS-fed comparison. Tubulin polymerization-promoting protein family member 3 (TPPP3) is a protein that regulates microtubule dynamics [49], and Serine/threonine-protein kinase Sg1k (SGK1) is a kinase that regulates a variety of ion channels, transcription factors, cellular enzymes, cell growth, membrane transporters, and is known to play a significant role in the stress response [50,51,52,53]. SGK1, is a negative regulator of VEGF and BDNF, has been shown to interact with NF-KB, RAN, mTOR, FOXO3A, and is increased in a human hippocampal progenitor cell line during MDD, and decreased in the prefrontal cortex of PTSD patients [54,55,56]. Licznerski et al. (2015) [56] also found that in the hippocampus of foot-shock stressed rats, SGK1 mRNA was underexpressed, but the amount of hippocampal protein remained unchanged. Zhang et al. (2016) [57] found that in a chronic corticosterone (CORT) mouse model for anxiety and depression, glucocorticoid receptor levels were diminished, leading to an insufficient hippocampal neurogenesis. By treating mice with baicalin, they were able to restore hippocampal neurogenesis and reverse depression-like behaviours [57]. In their model, baicalin is thought to be undoing phosphorylation of SGK1, allowing it to phosphorylate the glucocorticoid receptor, encouraging translation to the nucleus, where it may promote neurogenesis.

### 3.3. JB-1-Modulated Genes in the Blood

Several mRNAs were upregulated in the blood of JB1-fed mice compared to both LR6475 and PBS-fed mice. They were: FKBP1A, SNRPN, CELF4, GPM6B, APBB1, NCDN, RUNDC3A, CPE, and CLSTN1.

Neuronal membrane glycoprotein gene (GPM6B) codes for a protein involved in bone formation and osteoblast function, as well as being a binding partner of the serotonin transporter (SERT) [58,59]. It has been suggested that GPM6B could play a role in regulating SERT cellular trafficking and activity, which could potentially have a broad impact on mood disorders [59,60]. It has also been found that GPM6B expression is severely reduced in the hippocampus of depressed suicides, and mechanistically, it has been proposed that this lack of GPM6B in the hippocamus alters oligodendrocyte function to contribute to MDD [61,62]. 

Carboxypeptidase E (CPE) codes for an exopeptidase that removes C-terminal lysine or arginine acids from peptides [63]. Rodriguiz et al. (2013) [63] found that a point mutation to the CPE gene induced anxiety-like behaviours in older mice, and depression-like behaviours in mice of all ages. Anxiety-like behaviours were reversed following acute treatment with fluoxetine or diazapam, while depression-like behaviours were reversed with acute reboxetine administration, or prolonged treatment with bupropion or fluoxetine [63]. A similar mutation was discovered in an Alzheimer’s patient by Cheng et al. (2016) [64] and replicating the mutation in mice led to decreased neurogenesis in the hippocampus, decreased dendrites, impaired memory, and depression-like behaviour.

Calsyntenin-1 (CLSTN1) codes for a protein that encourages vesicle association with KLC1 in axonal anterograde transport [65]. CLSTN1 has previously been found to be differentially hypermethylated in the blood of MDD patients (*n* = 118) compared to healthy subjects (*n* = 236) [66]. The mechanism by which CLSTN1 affects depression has yet to be elucidated, however Li et al. (2021) [67] confirmed that overexpression of CLSTN1 in the hippocampus of mice and rats increased anxiety and depression-like phenotypes. We did not find that JB1 altered hippocampal expression of CLSTN1, and it is interesting that we see it increased in the blood. As the differentially methylated DNA samples in Davies et al. (2014) [66] were collected from the blood, this suggests that a dearth of CLSTN1 expression in the body is implicated in depression.

## 4. Materials and Methods

### 4.1. Laboratory Methods

#### 4.1.1. Feeding and Tissue Collection

7–9-week-old male balb/c mice from Charles River Laboratories were orally gavaged with 200 µL of either *Lacticaseibacillus rhamnosus* JB-1 (2 × 10⁹), *Limosilactobacillus reuteri* 6475 (2 × 10⁹), or PBS (*n* = 5, 5, 5). Mice were gavaged once per day for 2 weeks and sacrificed 3 h after the last gavage. Trunk blood was collected and whole brains were flash frozen and stored. Later, hippocampi of alternating side half brains were isolated and stored for RNA isolation.

#### 4.1.2. RNA Isolation and Analysis

Total RNA was isolated from fresh whole blood using a PureLink RNA mini kit for total RNA isolation and using the manufacturer-recommended protocol for whole blood extraction.

Total RNA from hippocampi was isolated first by homogenizing the tissue with mortar and pestle in lysis buffer, followed by up-down pipetting through a 27-gauge syringe. After the tissue was completely homogenized, the same PureLink RNA mini kit was used to extract total RNA using the manufacturer-recommended protocol for tissue extraction.

Four samples from each gavage group, totaling at 12 samples for blood and hippocampus, underwent paired-end RNAseq (P3, 2 × 50 bp) on an illumina NextSeq for mRNA discovery and analysis.

### 4.2. Bioinformatic and Statistical Analysis

#### 4.2.1. Data Preprocessing and Differential Expression

Raw RNAseq data were adaptor trimmed and aligned using the ‘RNA-Seq Alignment’ app in Illumina BaseSpace which uses the Spliced Transcripts Alignment to a Reference (STAR) alignment method with the USCS mm10 refseq gene annotation file [68]. Next transcript expression of the annotation data was quantified by Salmon to produce count data [69] within the ‘RNA-Seq Alignment’ app, variant calling was performed by Strelka Variant caller [70], quality control metrics were performed by picard. Raw count data were downloaded, and differential expression was performed by using ‘DESeq2’ package in R [71], which automatically filters low expressed genes and normalizes the data by the geometric mean. Individual mRNAs were considered to be differentially expressed when they exhibited adjusted *p*-value < 0.05, and an |FC| > 1.5. *p*-values were adjusted using Benjamini–Hochberg method [72].

#### 4.2.2. PCA

Percent variables and principal components were calculated using DESeq2′s plotPCA function on the list of internally normalized and filtered genes, and then graphed using ggplot2 [73].

#### 4.2.3. Enrichment Analysis

Fold change and adjusted *p*-values values generated by DESeq2′s ‘results’ function were used by the generally applicable *gage* (gene-set enrichment for pathway analysis) package in R to generate enrichment results to the KEGG and Hallmark pathway gene sets [74]. KEGG pathway visualization was performed using the ‘pathview’ R package [75] and shows relative expression of each gene in each differentially expressed pathway (determined by gage). Enrichment of a certain pathway was considered significant at Benjamini–Hochberg adjusted *p*-value < 0.05.

## 5. Conclusions

Here, we identified several pathways and genes that may be associated with JB-1, treatment that may plausibly be related to the effects of these organisms on behavior, summarized in (Table 2 and Table 3). Some are likely related to the previously described immunomodulatory effects of these organisms. In particular, the role interferon signaling pathways in mediating gut–brain signaling warrants further exploration. The lack of any significant modified pathways or genes following LR6475 is surprising. However, this study examined the direct effect of JB-1 and LR6475 on normal BALB/c mice, while this mouse strain has high trait anxiety it may be that the effects of LR6475 would only be observable following a stress challenge or in a pathological mood disorder model.

There are certain limitations to the current study. The brains used in the study were not perfused following collection, and therefore could contain a small amount of blood from within capillaries in the hippocampus. Furthermore, only male mice were used, there is evidence that the outcome of gut–brain signaling can be sex dependent and thus sex comparisons would be worthy of examination in the future. This is particularly pertinent as in humans mental health disorders disproportionately affect women. Finally, WGCNA recommends 15 samples minimum for analysis and we performed the analysis with 12, which may have reduced the precision of the results.

Overall, our results highlight that microbes labeled as psychobiotics, or potential psychobiotics, induce complex changes in systemic gene expression which are far from uniform between organisms. A better understanding of the many pathways impacted by individual organisms may help develop more tailored microbe-based approaches to specific mental health issues.

## Figures and Tables

**Table 1:** Enriched pathways in JB-1 compared with PBS and compared with LR6475. The left column shows the relative direction of expression in JB-1 for each gene set and the right column shows the adjusted *p*-Value for each. Both JB-1 comparisons had nearly identical pathways and corresponding directions of expression, except for the last two entries on the table, which were significant only in the JB-1 vs. PBS comparison.

Enriched In	Pathway Name	Adj.*p*-Val (JvC, JvR)
JB-1	HALLMARK_OXIDATIVE_PHOSPHORYLATION	3.4 × 10^−4^, 3.4 × 10^−4^
JB-1	KEGG_OXIDATIVE_PHOSPHORYLATION	3.7 × 10^−3^, 9.9 × 10^−3^
JB-1	KEGG_PARKINSONS_DISEASE	1.3 × 10^−2^, 2.3 × 10^−2^
JB-1	KEGG_HUNTINGTONS_DISEASE	1.3 × 10^−2^, 2.3 × 10^−2^
JB-1	KEGG_ALZHEIMERS_DISEASE	2.1 × 10^−2^, 3.7 × 10^−2^
PBS	HALLMARK_ALLOGRAFT_REJECTION	1.5 × 10^−6^, 2.7 × 10^−6^
PBS	HALLMARK_INTERFERON_GAMMA_RESPONSE	5.4 × 10^-6^, 8.9 × 10^-6^
PBS	HALLMARK_INFLAMMATORY_RESPONSE	4.6 × 10^−3^, 9.4 × 10^−3^
PBS	HALLMARK_INTERFERON_ALPHA_RESPONSE	7.7 × 10^−3^, 8.3 × 10^−3^
PBS	HALLMARK_TNFA_SIGNALLING_VIA_NFKB	3.2 × 10^−2^, 3.1 × 10^−2^
PBS	HALLMARK_IL6_JAK_STAT3_SIGNALLING	3.2 × 10^-2^, 2.5 × 10^-2^
PBS	KEGG_PRIMARY_IMMUNODEFICIENCY	1.5 × 10^−3^, 3.6 × 10^−3^
PBS	KEGG_CYTOKINE_CYTOKINE_RECEPTOR_INTERACTION	4.9 × 10^−3^, 9.2 × 10^−3^
PBS	KEGG_JAK_STAT_SIGNALLING_PATHWAY	4.9 × 10^−3^, 8.0 × 10^−3^
PBS	KEGG_CHEMOKINE_SIGNALLING_PATHWAY	1.8 × 10^−2^, 1.0 × 10^−2^
PBS	KEGG_HEMATOPOIETIC_CELL_LINEAGE	1.9 × 10^−2^, 1.0 × 10^−2^
PBS	KEGG_B_CELL_RECEPTOR_SIGNALLING_PATHWAY	1.9 × 10^−2^, 2.8 × 10^−2^
PBS	KEGG_RIBOSOME	2.1 × 10^−2^, 6.7 × 10^−3^
PBS	KEGG_T_CELL_RECEPTOR_SIGNALLING_PATHWAY	2.2 × 10^−2^, 2.8 × 10^−2^
PBS	KEGG_LEUKOCYTE_TRANSENDOTHELIAL_MIGRATION	3.8 × 10^−2^
PBS	KEGG_NATURAL_KILLER_CELL_MEDIATED_CYTOTOXICITY	4.5 × 10^−2^

**Table 2:** Differential expression of pathways relevant to depression, in depression, after established antidepressant treatment, and following JB-1 treatment in mice. The up and down arrows represent the direction of change in pathway expression in our findings and in literature.

Pathway	INF-γ/INF-α	JAK/STAT	TNF-α via NF-KB
Change in Depression	↑ generally increased, but suboptimal expression has also been associated with depression [33,76,77]	↑ activation in stress and depression in mice [78]	↑ in prefrontal cortex and hippocampus of mice [40]
Change in MDD Treatment	↓ after treatment with either sertraline, clomipramine, or trazodone in human blood [35]	↓ phosphorylation (activation) of Jak-3 returned to normal in mice following Amitriptyline treatment [78]	↓ SSRIs such as imipramine reduce TNF-α levels in rats [79]
Blood Change Following JB-1	↓ mRNA (5.4 × 10^−6^) (7.7 × 10^−3^)	↓ mRNA (4.9 × 10^−3^)	↓ mRNA (3.2 × 10^−2^)

**Table 3:** Differential expression of genes relevant to depression, in depression, after established antidepressant treatment, and following JB-1 treatment in mice. The up and down arrows represent the direction of change in pathway expression in our findings and in literature.

Gene	SGK1	GPM6B	NCDN	CLSTN1
Change in Depression	↓ in hippocampus of rats [56]	↓ in the hippocampus of humans [60]	↓ in CNS of mice [80]	↓ in blood of humans [66], ↑ in hippocampus of rats and mice [67]
Change in Depression Treatment	↑ mRNA in hippocampus and prefrontal cortex of rats following icariin or baicalin treatment [81,82]	N/A	↓ in hippocampus after ketamine treatment in rats [83]	N/A
Change Following JB-1	↑ mRNA in hippocampus of mice (4.5 × 10^−2^)	↑ mRNA in blood of mice (7.6 × 10^−4^)	↑ mRNA in blood of mice (8.5 × 10^−3^)	↑ mRNA in blood of mice (1.6 × 10^−2^)

## Data Availability

Not applicable.

## Supplementary Materials

The following supporting information can be downloaded at: https://www.mdpi.com/article/10.3390/ijms23179653/s1.
