# Supplementary figures and images for: Effects of Two Distinct Psychoactive Microbes, Lacticaseibacillus rhamnosus JB-1 and Limosilactobacillus reuteri 6475, on Circulating and Hippocampal mRNA in Male Mice
Source: Int J Mol Sci. 2022 Aug 25;23(17):9653. doi: 10.3390/ijms23179653 (PMC9456087; doi:10.3390/ijms23179653)

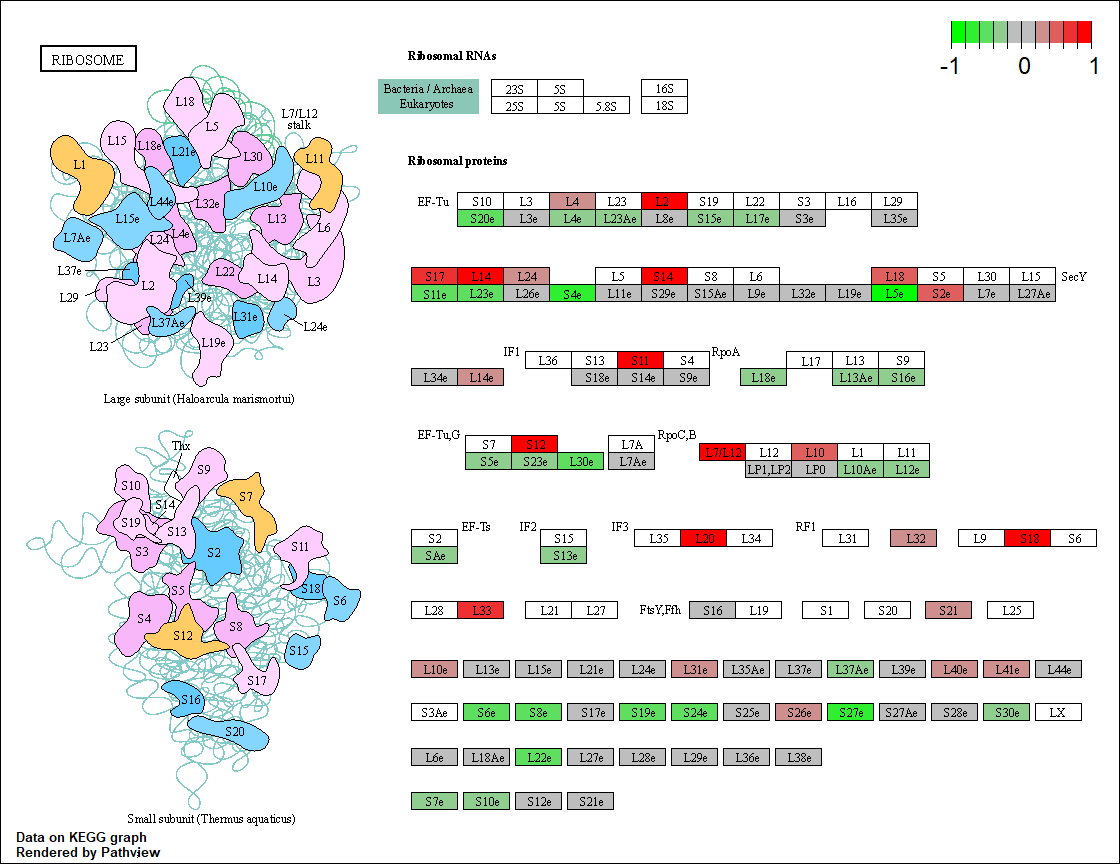

Supplement: Supplementary file 1 [file ijms-23-09653-s001.zip › Supplementary Figure S1.png]

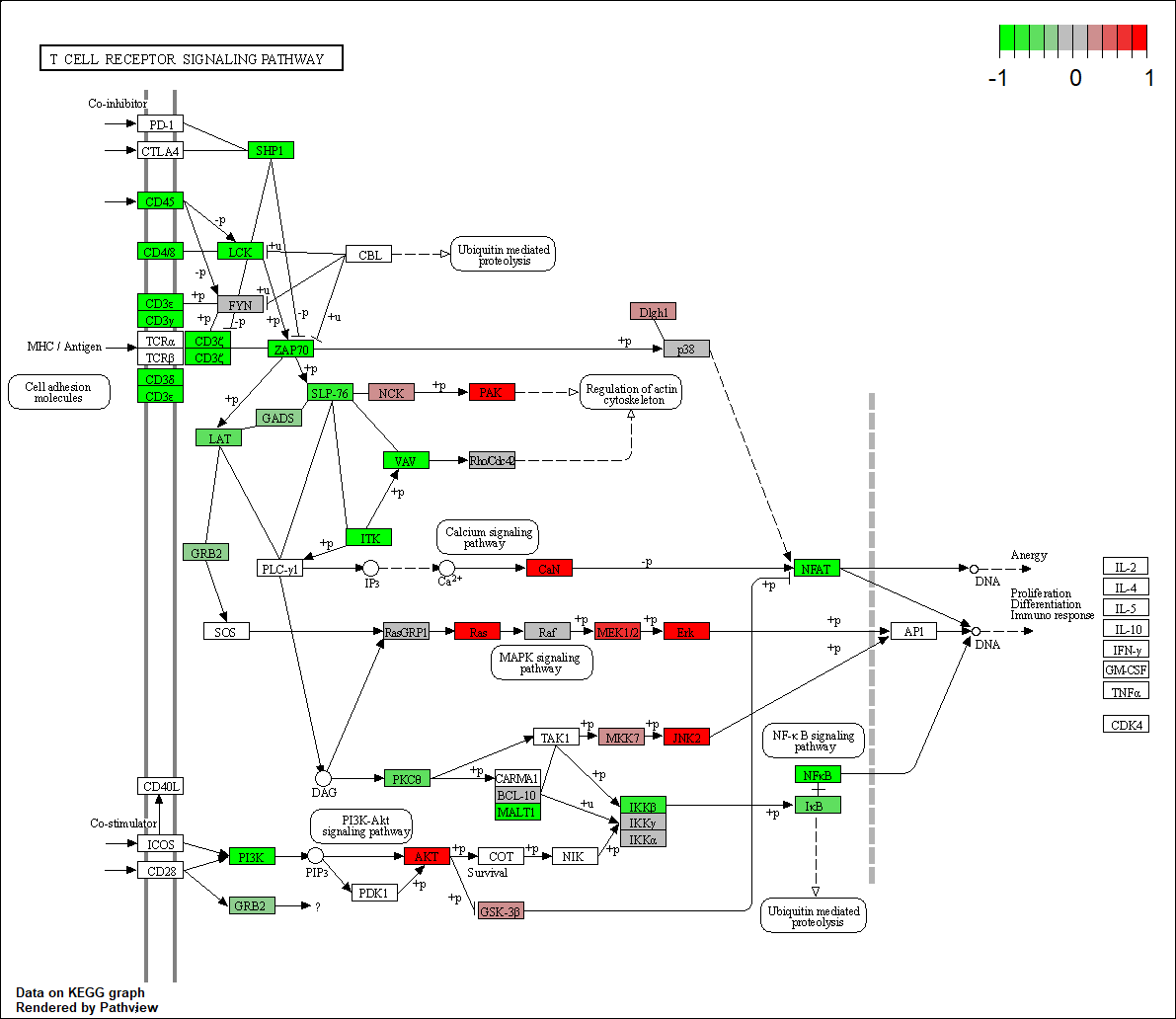

Supplement: Supplementary file 1 [file ijms-23-09653-s001.zip › Supplementary Figure S10.png]

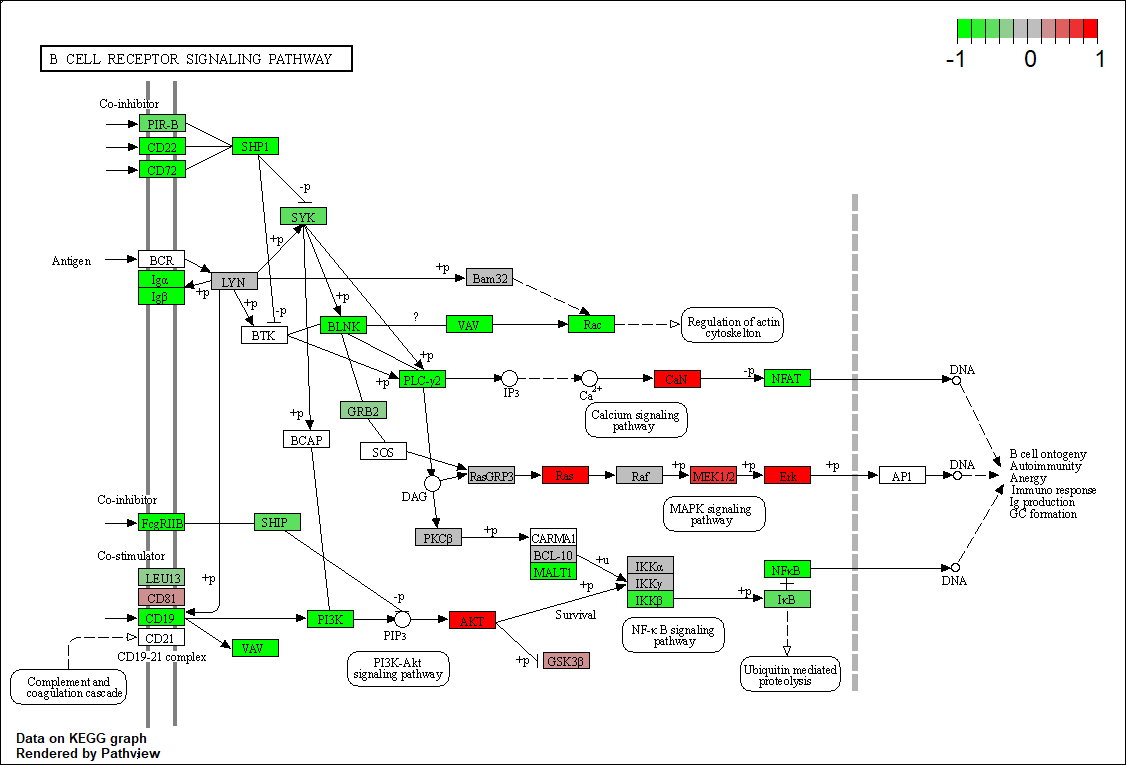

Supplement: Supplementary file 1 [file ijms-23-09653-s001.zip › Supplementary Figure S11.png]

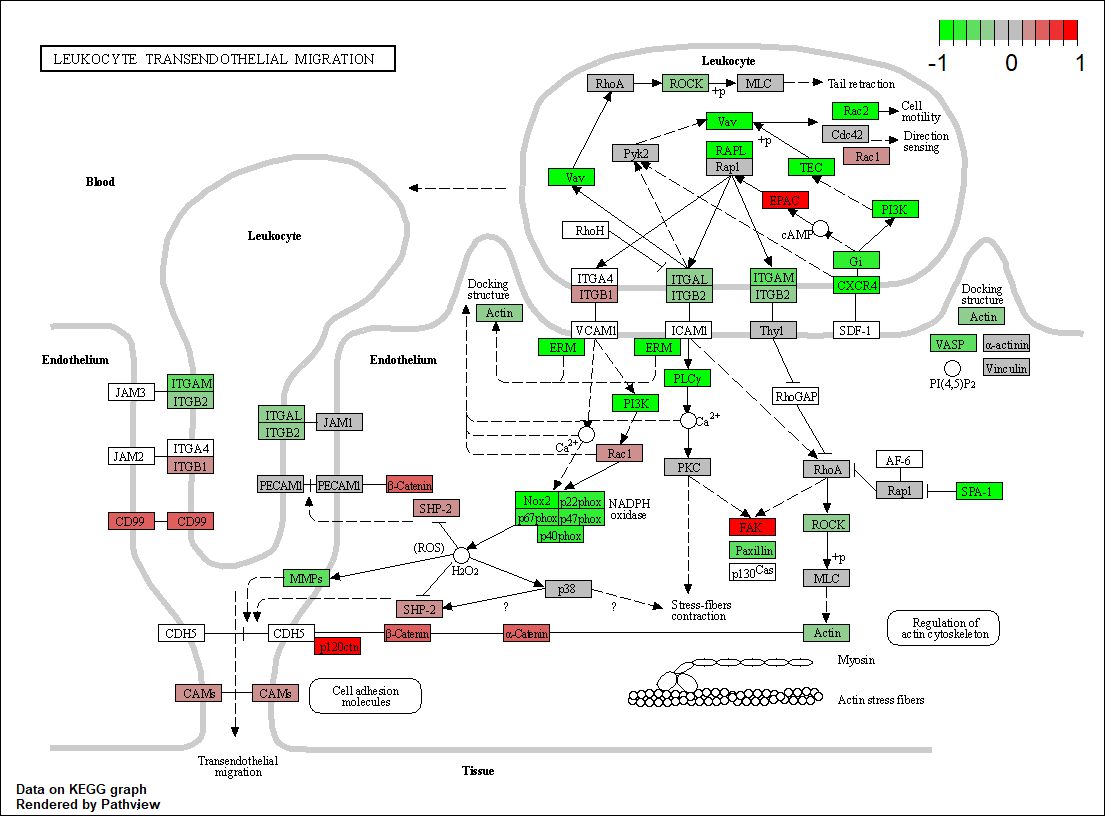

Supplement: Supplementary file 1 [file ijms-23-09653-s001.zip › Supplementary Figure S12.png]

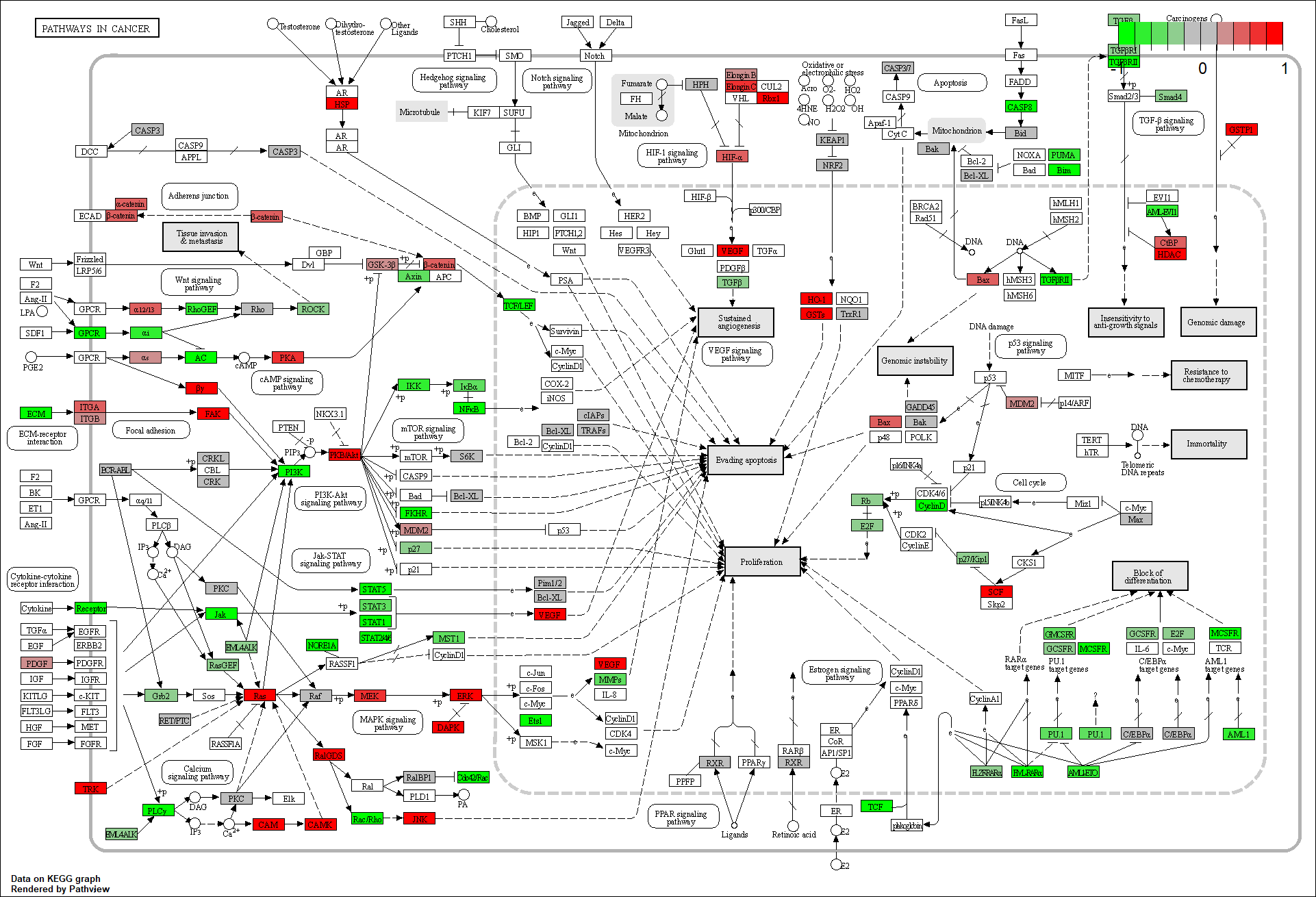

Supplement: Supplementary file 1 [file ijms-23-09653-s001.zip › Supplementary Figure S13.png]

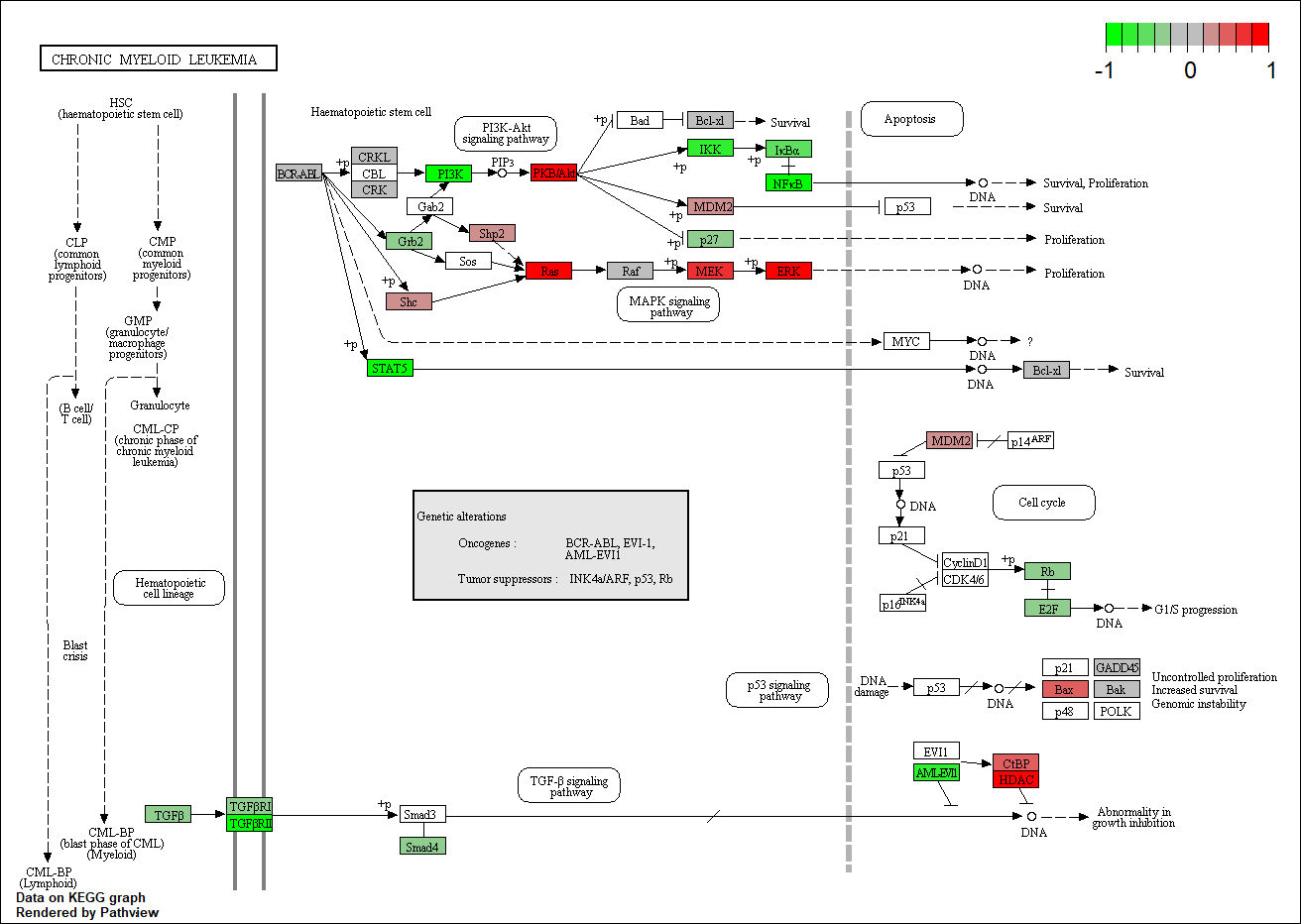

Supplement: Supplementary file 1 [file ijms-23-09653-s001.zip › Supplementary Figure S14.png]

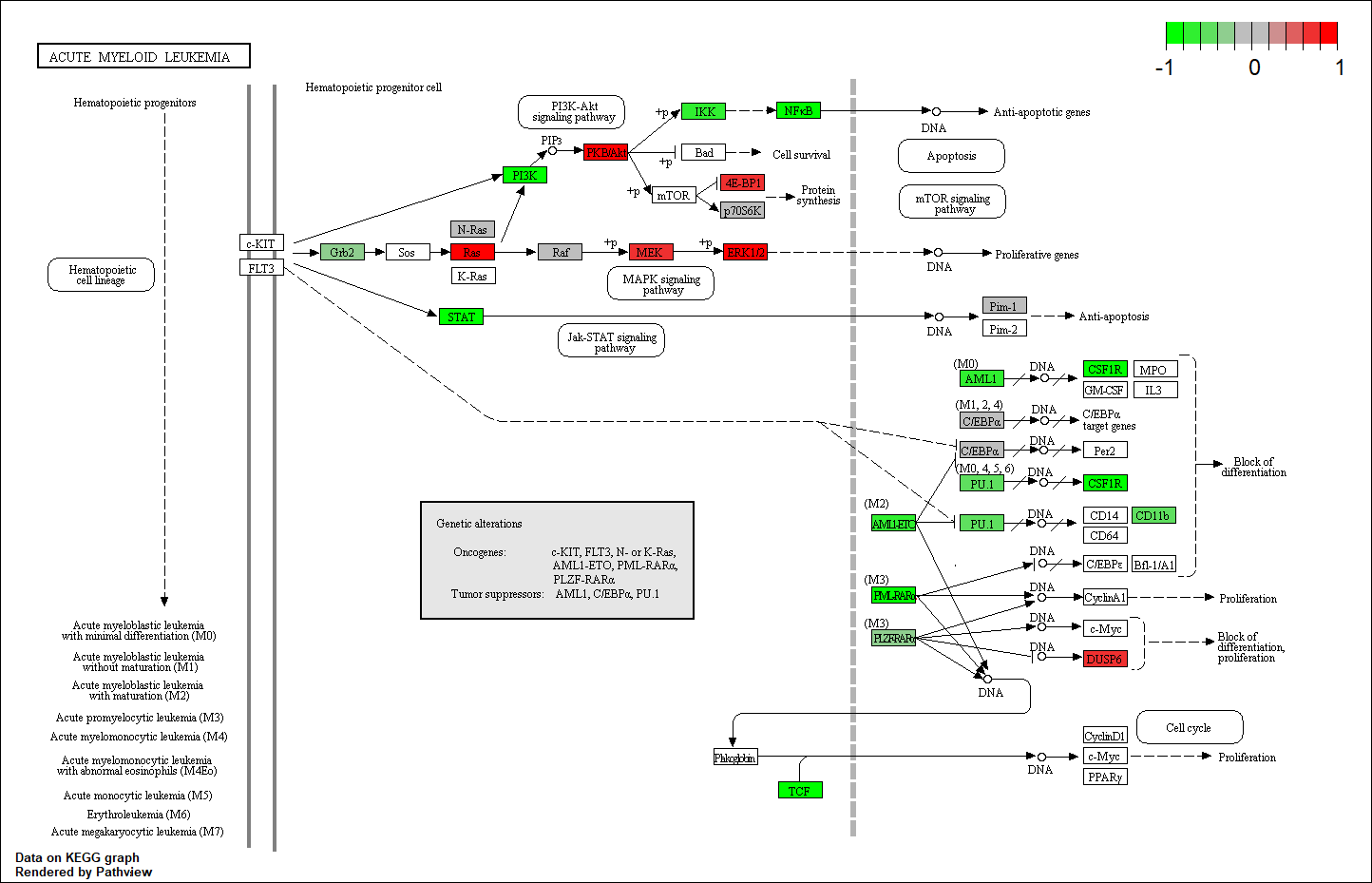

Supplement: Supplementary file 1 [file ijms-23-09653-s001.zip › Supplementary Figure S15.png]

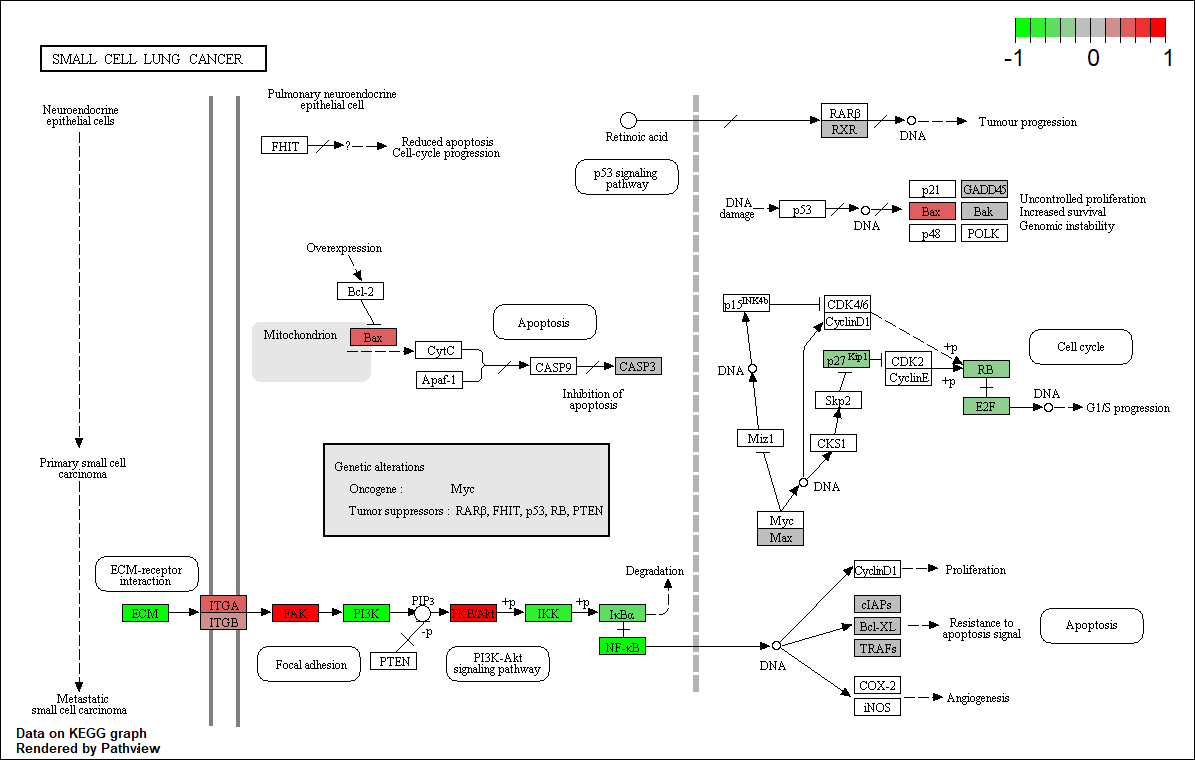

Supplement: Supplementary file 1 [file ijms-23-09653-s001.zip › Supplementary Figure S16.png]

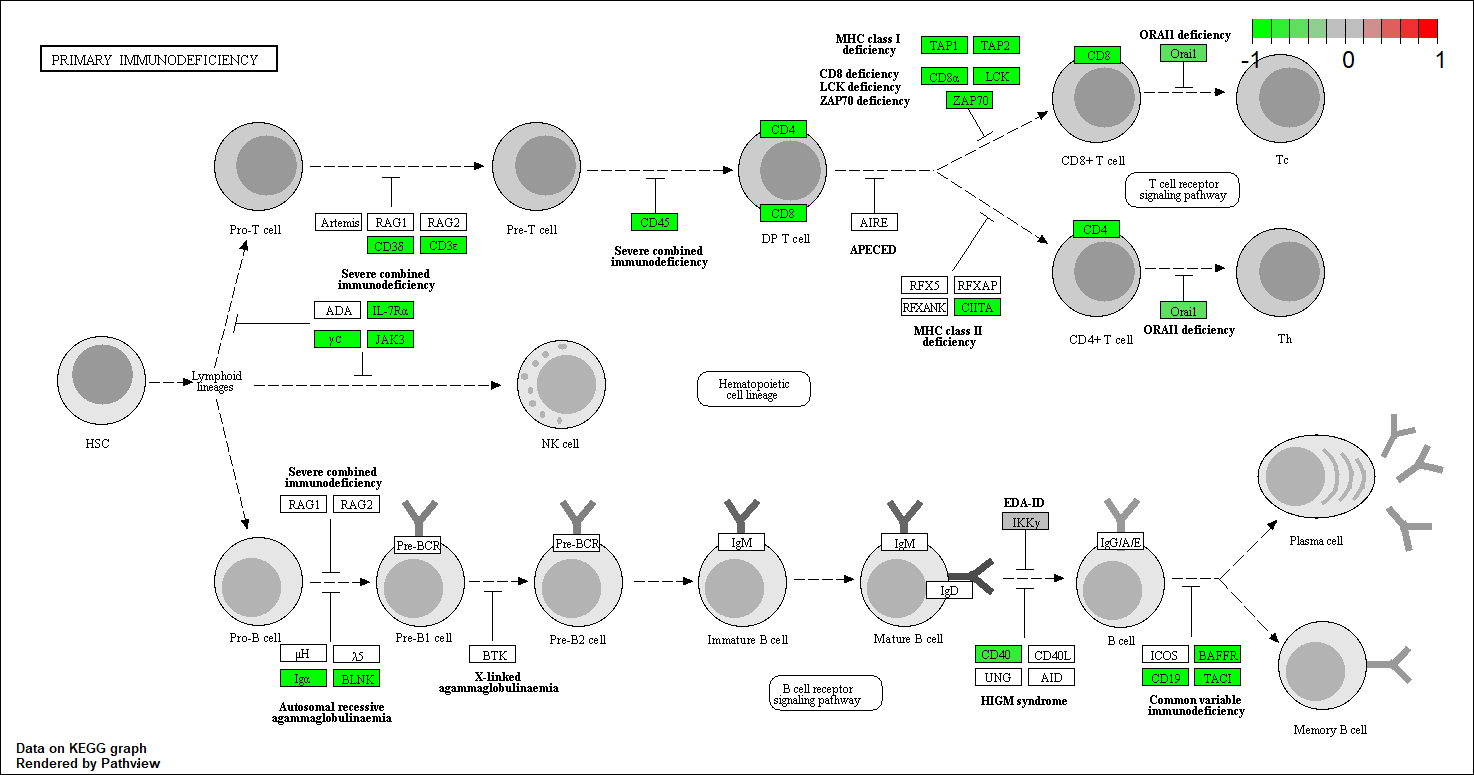

Supplement: Supplementary file 1 [file ijms-23-09653-s001.zip › Supplementary Figure S17.png]

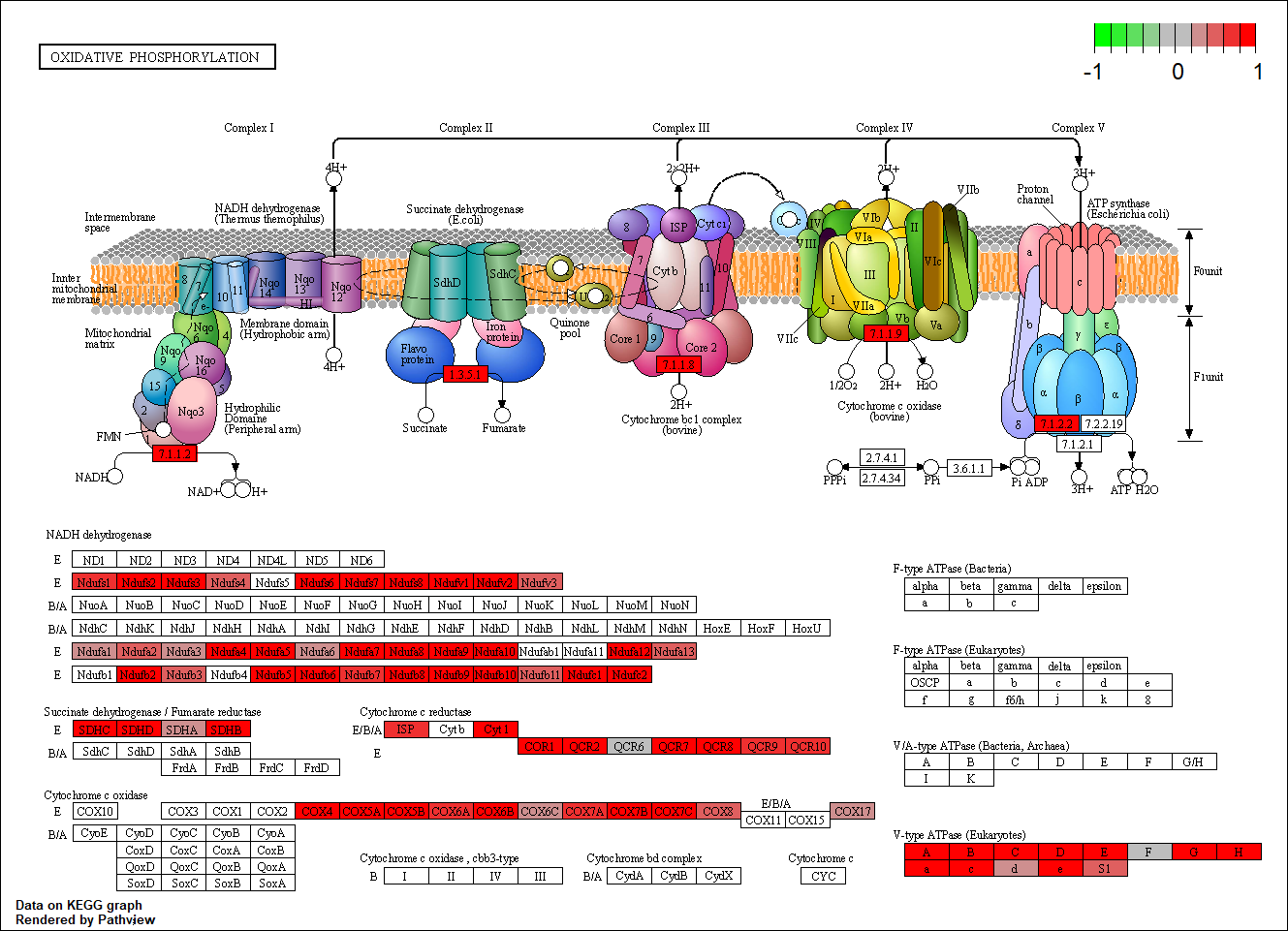

Supplement: Supplementary file 1 [file ijms-23-09653-s001.zip › Supplementary Figure S18.png]

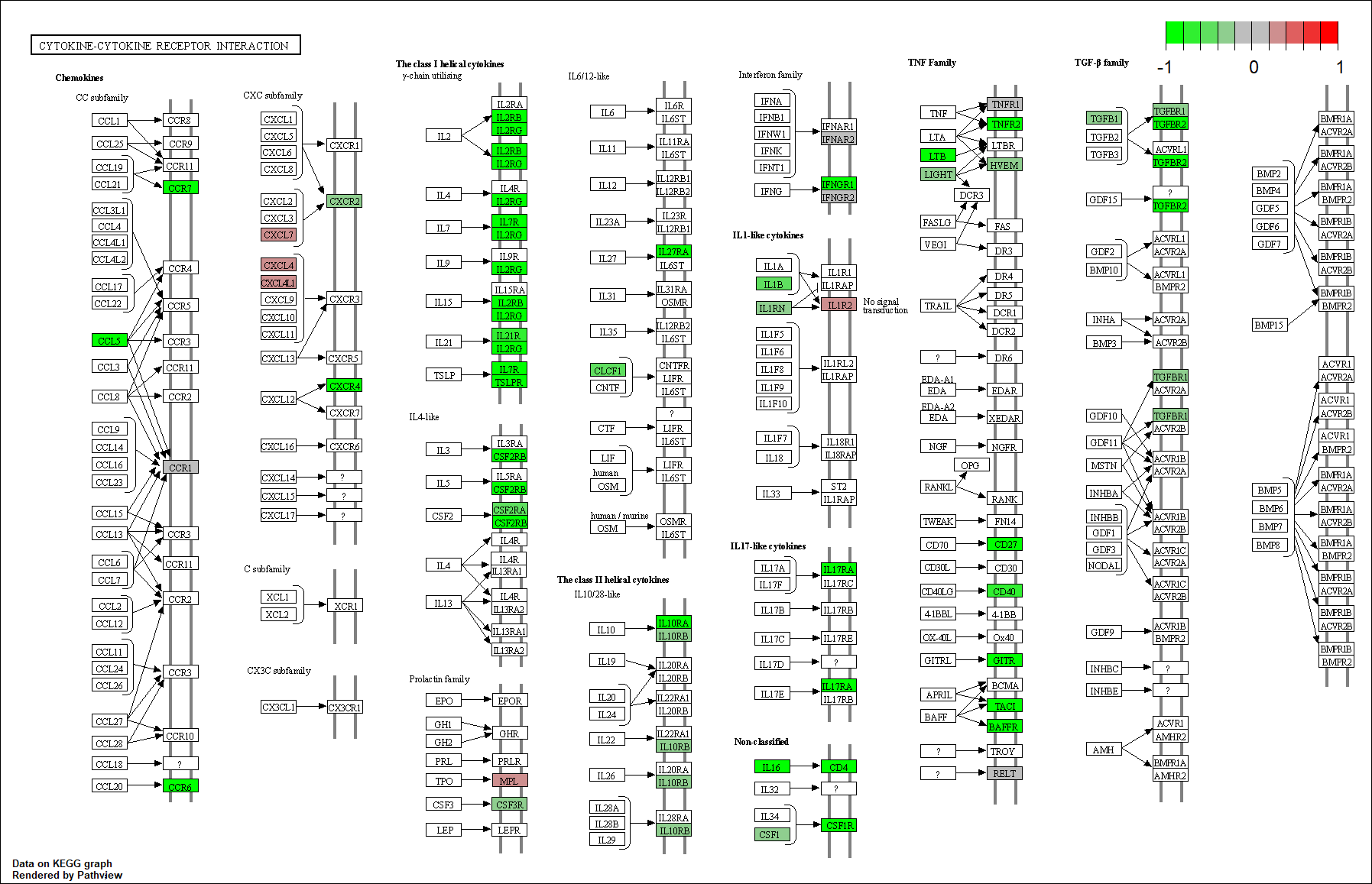

Supplement: Supplementary file 1 [file ijms-23-09653-s001.zip › Supplementary Figure S2.png]

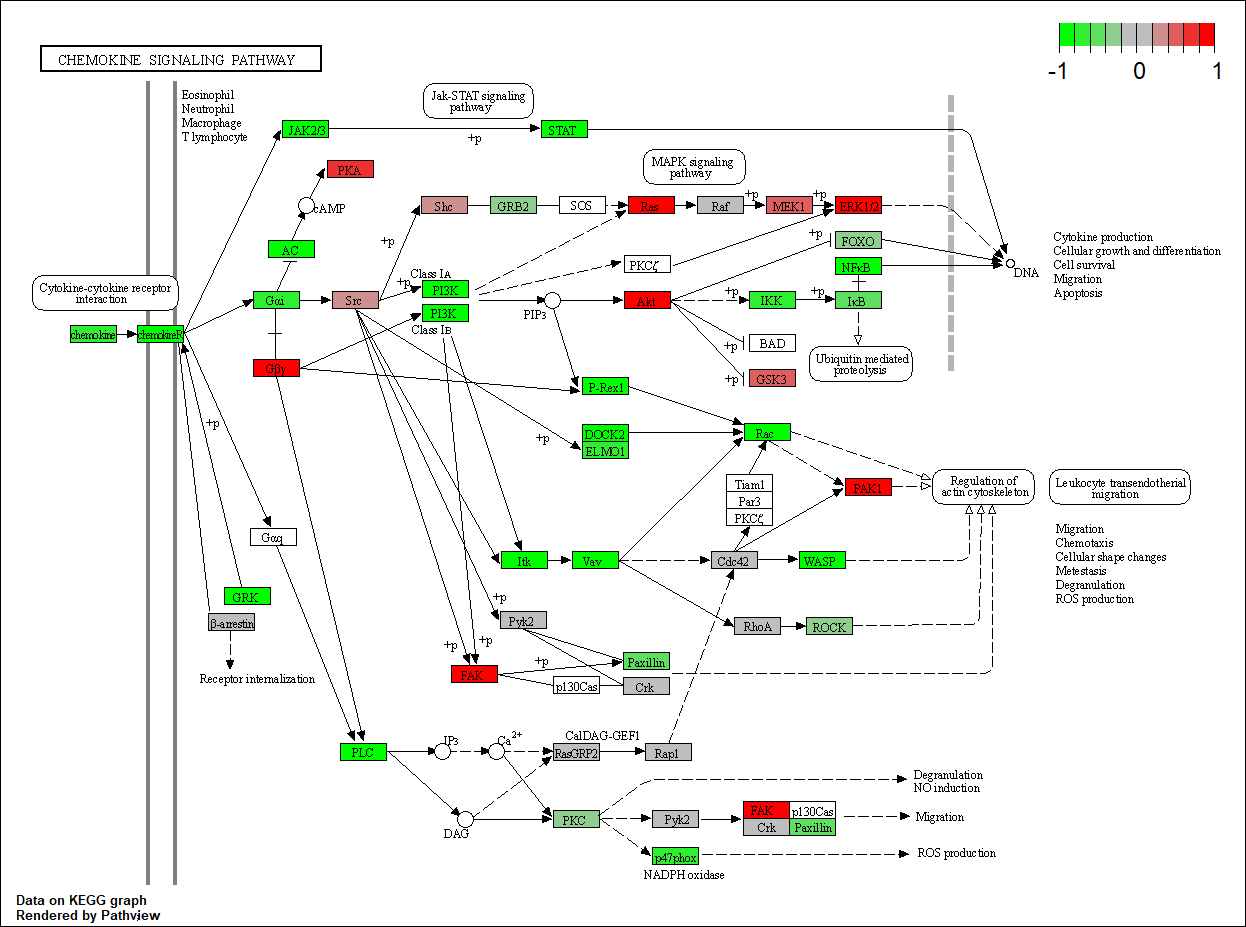

Supplement: Supplementary file 1 [file ijms-23-09653-s001.zip › Supplementary Figure S3.png]

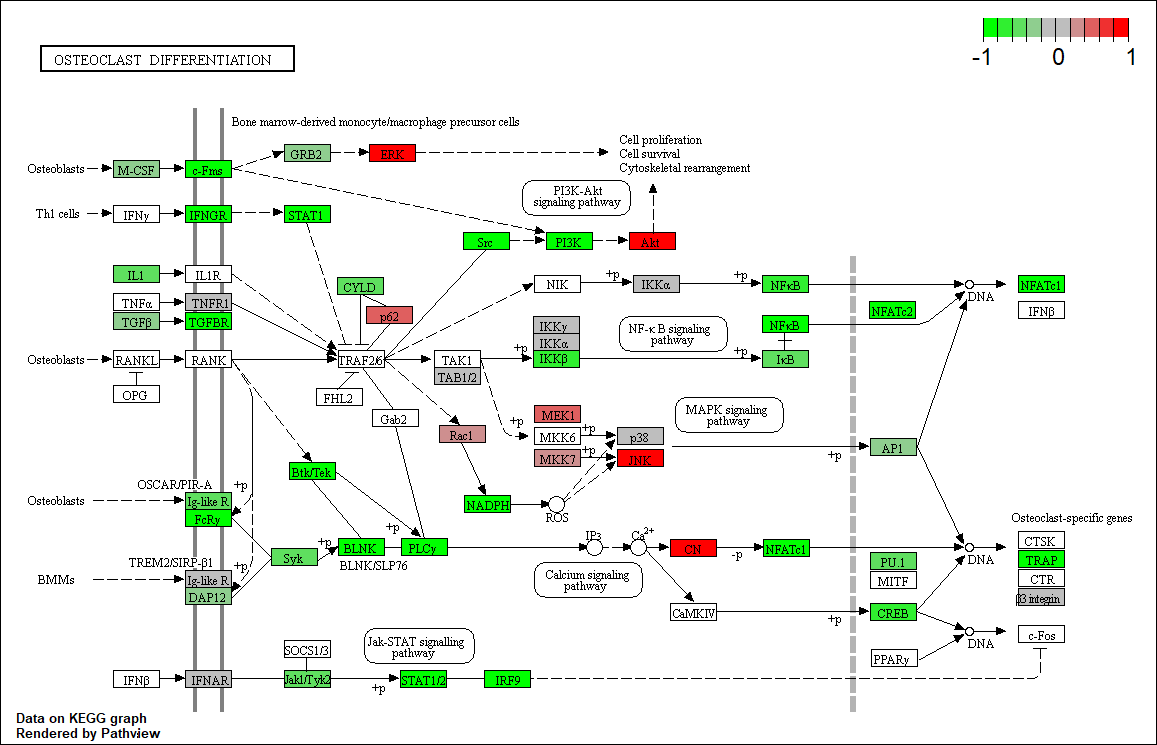

Supplement: Supplementary file 1 [file ijms-23-09653-s001.zip › Supplementary Figure S4.png]

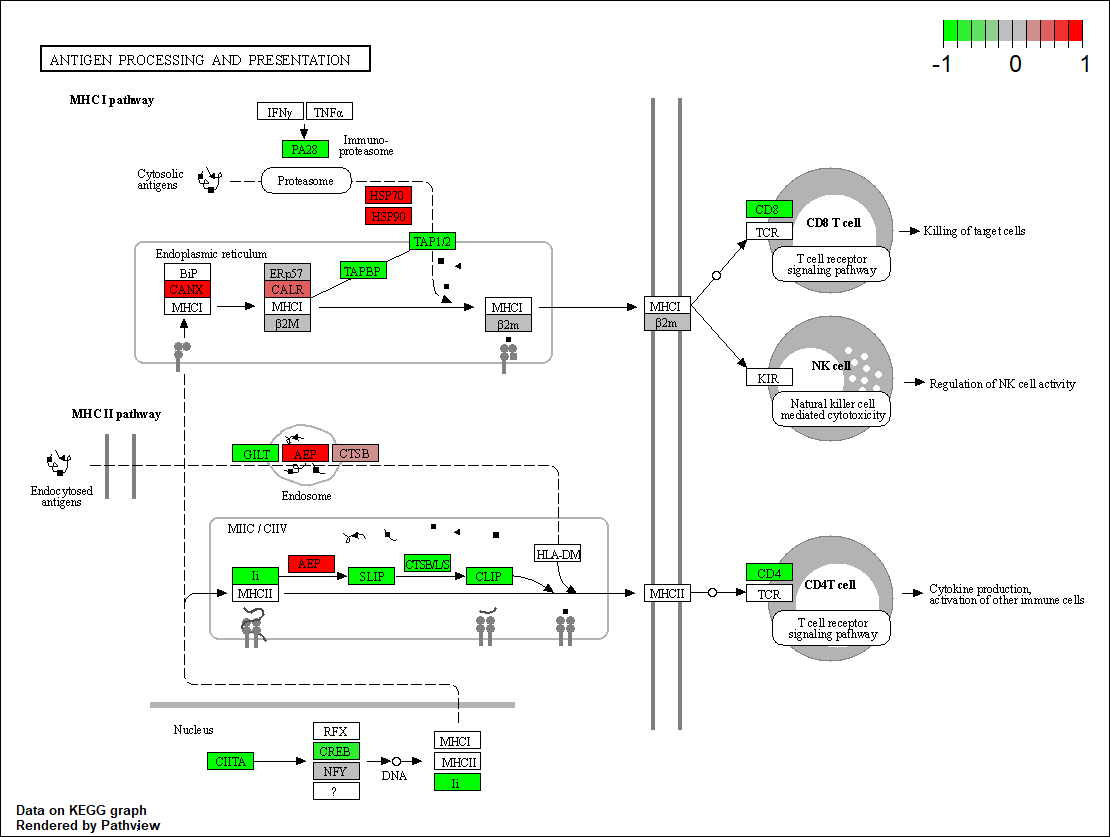

Supplement: Supplementary file 1 [file ijms-23-09653-s001.zip › Supplementary Figure S5.png]

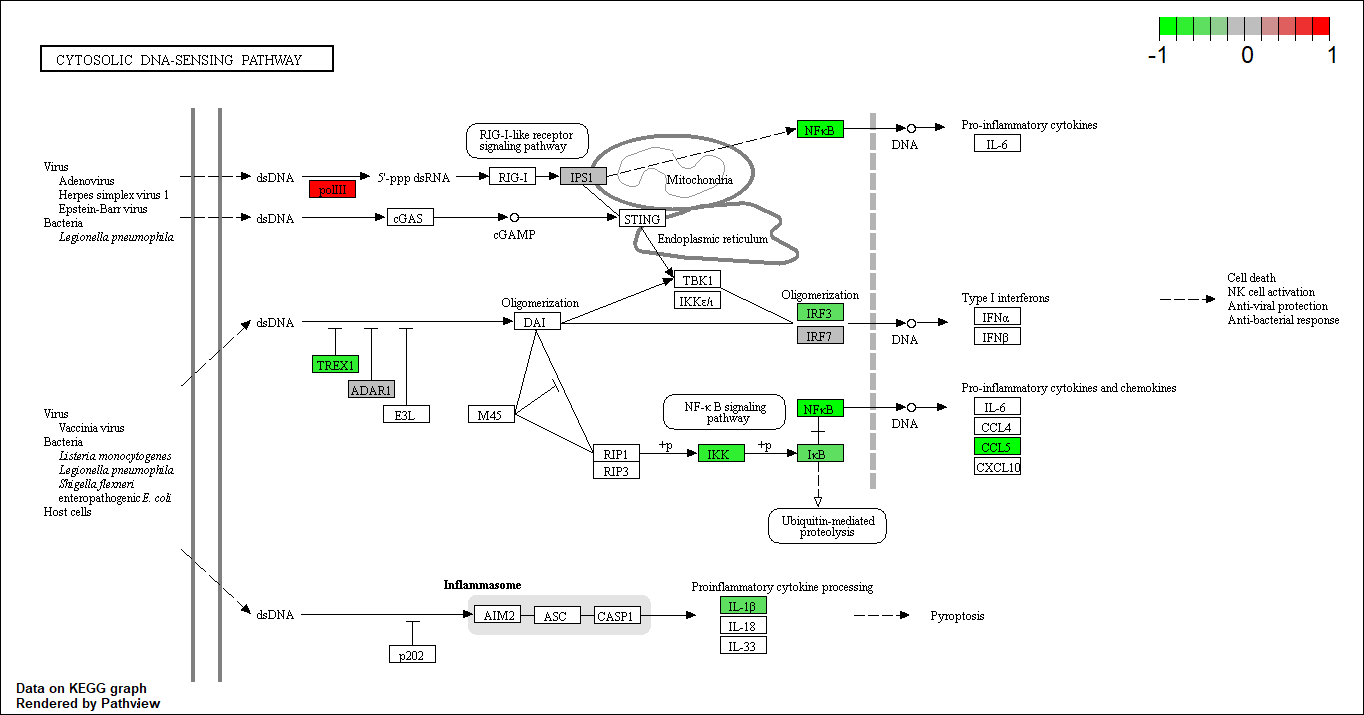

Supplement: Supplementary file 1 [file ijms-23-09653-s001.zip › Supplementary Figure S6.png]

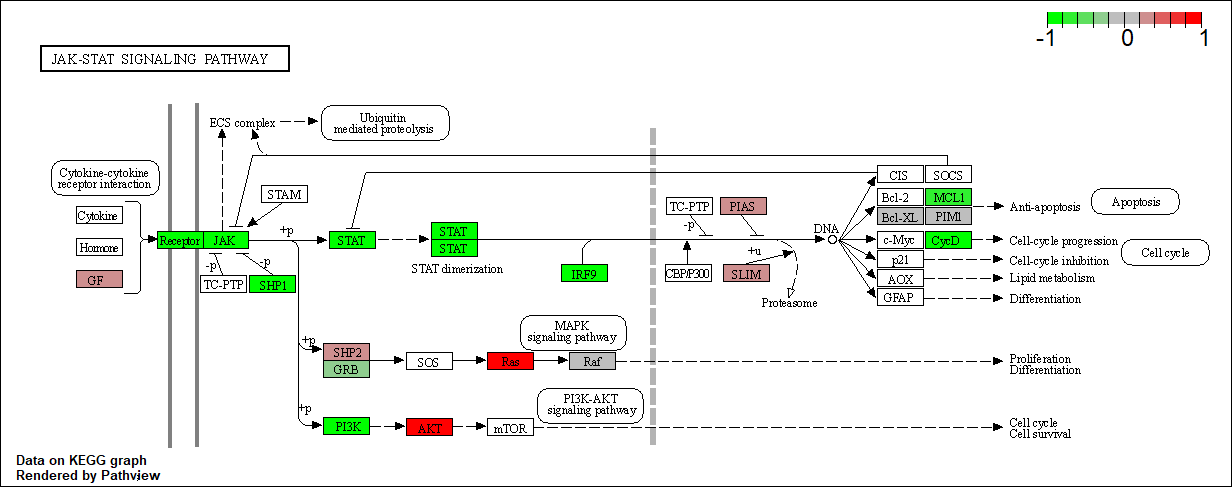

Supplement: Supplementary file 1 [file ijms-23-09653-s001.zip › Supplementary Figure S7.png]

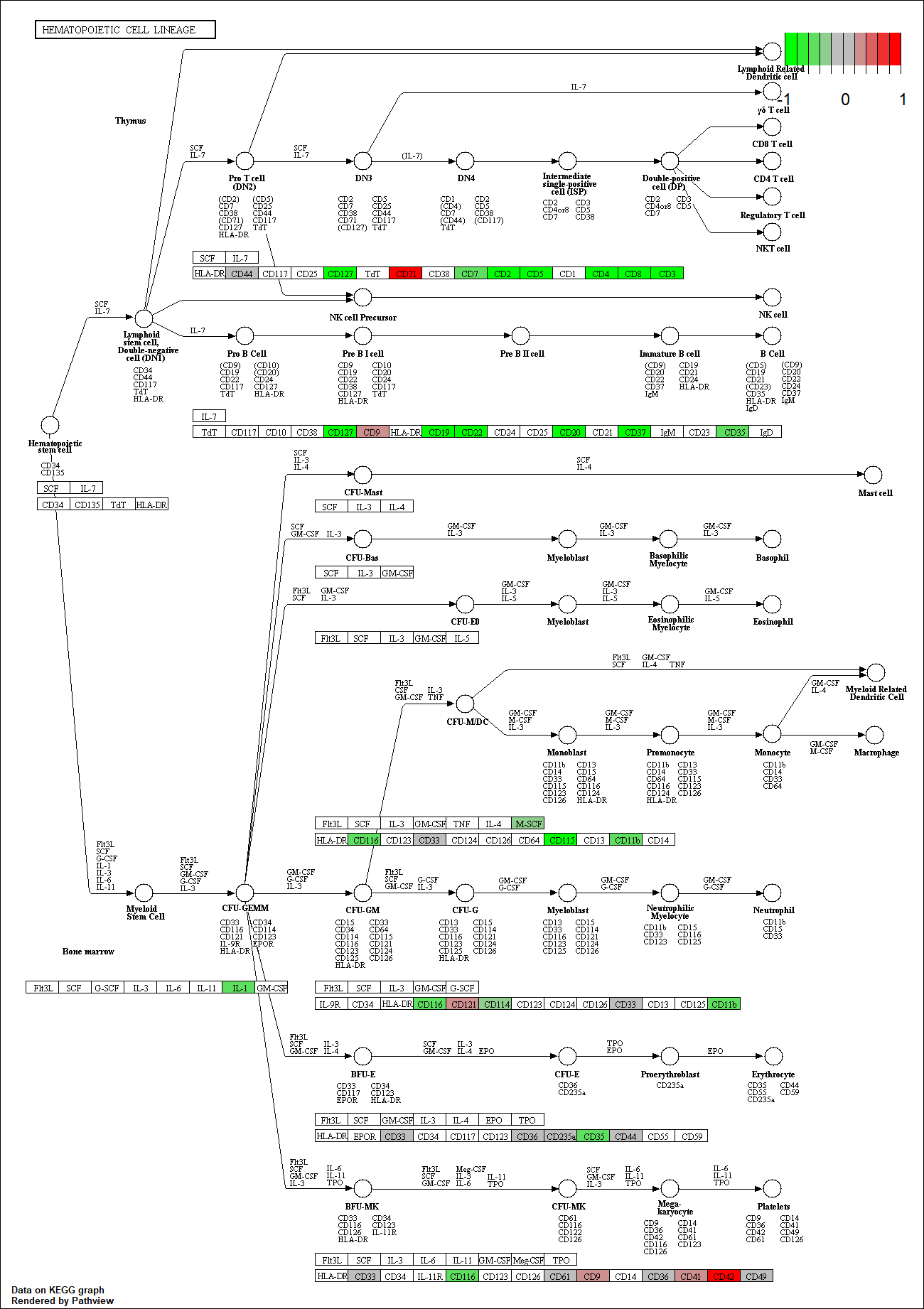

Supplement: Supplementary file 1 [file ijms-23-09653-s001.zip › Supplementary Figure S8.png]

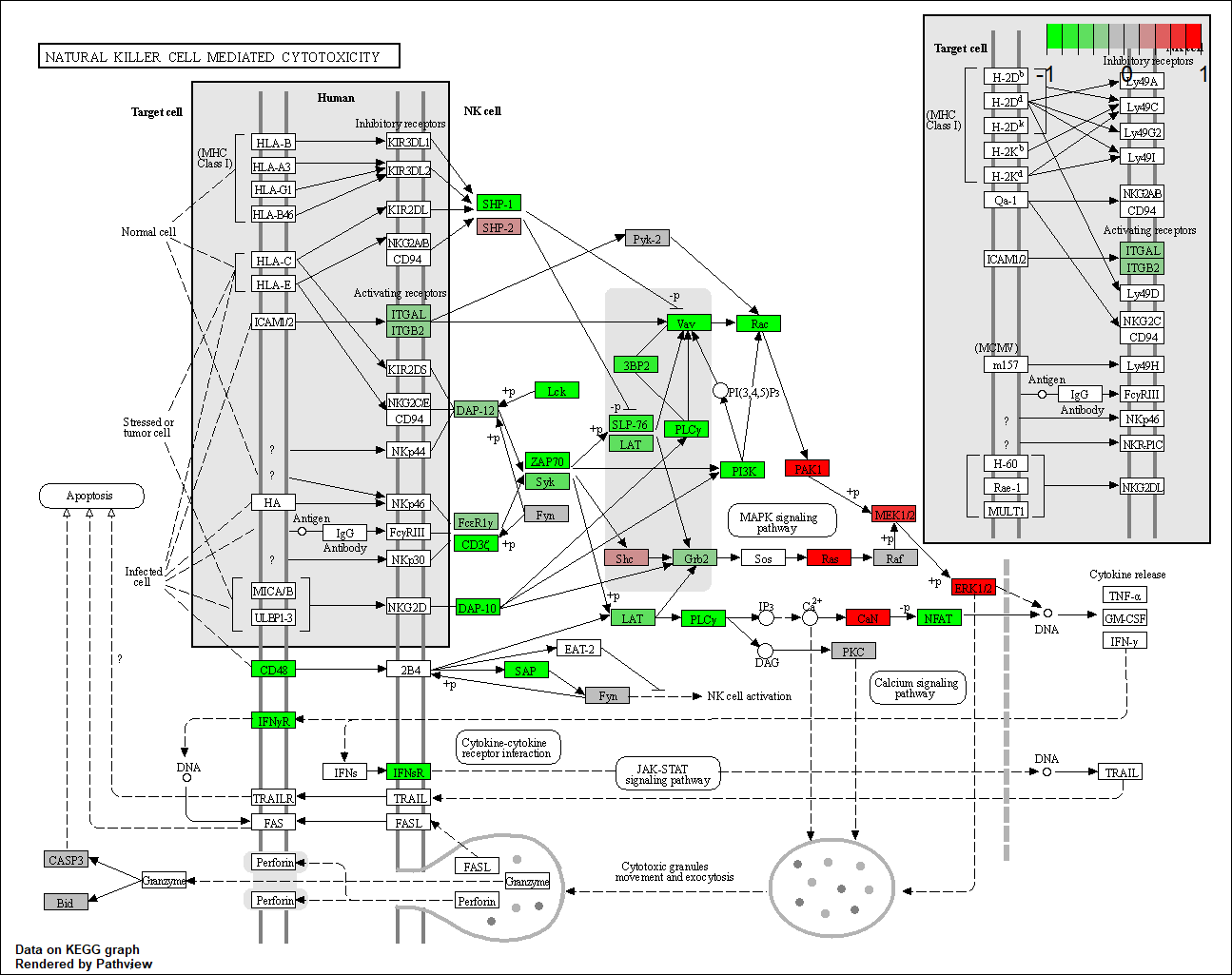

Supplement: Supplementary file 1 [file ijms-23-09653-s001.zip › Supplementary Figure S9.png]
